# Bundles of Brain Microtubules Generate Electrical Oscillations

**DOI:** 10.1038/s41598-018-30453-2

**Published:** 2018-08-09

**Authors:** María del Rocío Cantero, Cecilia Villa Etchegoyen, Paula L. Perez, Noelia Scarinci, Horacio F. Cantiello

**Affiliations:** 1 Laboratorio de Canales Iónicos, Instituto Multidisciplinario de Salud, Tecnología y Desarrollo (IMSaTeD, UNSE-CONICET), Santiago del Estero, Argentina; 2 Segunda Cátedra de Farmacología, Facultad de Medicina, UBA, Buenos Aires, Argentina

## Abstract

Microtubules (MTs) are long cylindrical structures of the cytoskeleton that control cell division, intracellular transport, and the shape of cells. MTs also form bundles, which are particularly prominent in neurons, where they help define axons and dendrites. MTs are bio-electrochemical transistors that form nonlinear electrical transmission lines. However, the electrical properties of most MT structures remain largely unknown. Here we show that bundles of brain MTs spontaneously generate electrical oscillations and bursts of electrical activity similar to action potentials. Under intracellular-like conditions, voltage-clamped MT bundles displayed electrical oscillations with a prominent fundamental frequency at 39 Hz that progressed through various periodic regimes. The electrical oscillations represented, in average, a 258% change in the ionic conductance of the MT structures. Interestingly, voltage-clamped membrane-permeabilized neurites of cultured mouse hippocampal neurons were also capable of both, generating electrical oscillations, and conducting the electrical signals along the length of the structure. Our findings indicate that electrical oscillations are an intrinsic property of brain MT bundles, which may have important implications in the control of various neuronal functions, including the gating and regulation of cytoskeleton-regulated excitable ion channels and electrical activity that may aid and extend to higher brain functions such as memory and consciousness.

## Introduction

MTs are unique components of the cellular cytoskeleton that form a wide variety of intracellular superstructures^1^. Highly polarized cells such as neurons, for example, present two structurally and functionally distinct domains, namely a single long, thin axon and multiple shorter dendrites that either transmit or receive electrical signals, respectively. MT stability is at the center of the polarization process of neurons, which is fundamental to their development and plasticity, as well as the development of neurodegenerative diseases. MTs form dense parallel arrays known as bundles in axons and dendrites, which are required for the growth and maintenance of neurites in neurons^2–4^. The organization of MT bundles in axons and dendrites largely depends on the prevalent type of MT-associated proteins prevalent in them. MAP2, for example is mainly found in dendrites while tau is mainly found in axons^3,5^. Another important aspect of MT organization and function relies on their remarkable and not as well understood biophysical properties. MTs are highly charged electrically polarized polymers, where their αβ tubulin heterodimeric units have a high electric dipole moment^6^, rendering these structures highly sensitive to electric fields both *in vitro*^7,8^ and *in vivo*^9^. Large uncompensated charge^10^ present in MTs likely plays an important role in electrostatic interactions implicated in MT-macromolecular complexing^11^. MTs are thought to generate oscillatory electric fields at expense of elasto-electrical vibrations^12^, which may explain our findings that electrically-stimulated MTs behave as biological transistors behaving as sophisticated nonlinear transmission lines, capable of supporting the amplification and axial transfer of electrical signals^13–18^. Within the cytoplasm MT-generated variable currents may contribute to the presence and modulation of large intracellular electric fields, which in turn, will help control cell function.

The above arguments support a potentially relevant role of electrical oscillations on brain MT bundles, which should be critical to neural function. We recently reported that MT sheets sustain electrical oscillations^19^, which are driven by a permanent electrical polarization from local asymmetries in the ionic distributions between the intra- and extra-MT environments. Thus, the MT wall behaves as an electrical oscillator that produces oscillatory ionic currents with variable amplitude and periodicity depending on the driving force and ionic compositions, and is consistent with the periodic on-off switching of the nanopores. In this context, here we explored whether rat brain MT bundles do have electrical properties consistent with electrical oscillators. Our findings indicate that rat brain MT bundles generate a wide variety of endogenous electrical oscillations. Interestingly, the cytoskeleton of cultured adult mouse hippocampal neurons also supported electrical oscillations establishing an electrical role for brain MTs.

## Results

**Figure 1:**
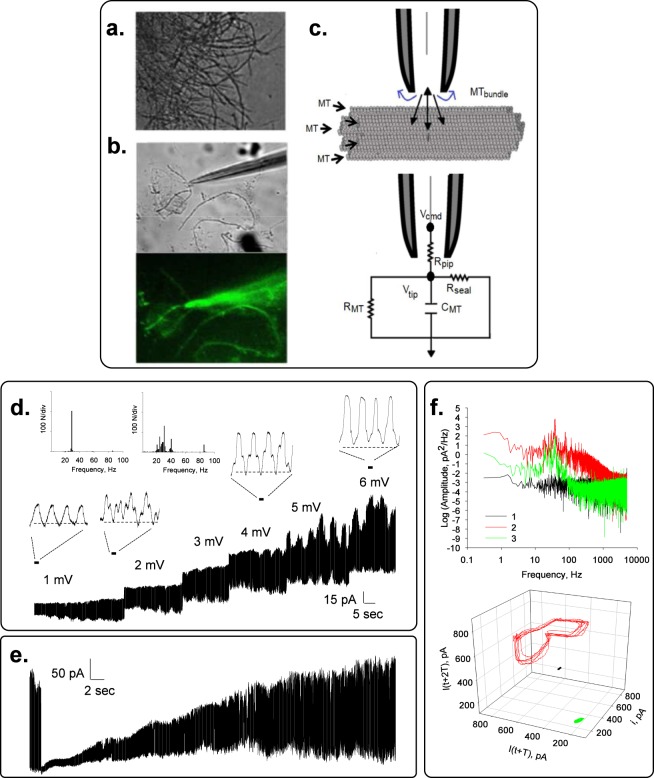
Experimental setup to study electrical oscillations of MT bundles. **(a**) Rat brain MT bundles obtained as reported in the Methods section (DIC x20). (**b)** *Top Panel*, DIC image of patch pipette approaching an MT bundle placed over a 2D MT sheet. *Bottom Panel*, Live immunochemical labeling of MTs by addition of both the anti-α ary antibodies to the preparation shown on *Top*. Please note that the loose patch connection between the MT bundle and the patch pipette allows backfilling of the pipette with both antibodies. (**c)** *Top Panel*, Schematics of the “loose-patch” clamp configuration to obtain electrical properties of MT bundles. The blue curved arrows from the pipette tip indicate leak currents that modify the amplitude of the holding potential applied from the amplifier. *Bottom Panel*, Schematic of the loose patch circuit applied to the MT surface. Resistances are shown for the pipette (*R*_*pip*_), patch surface (*R*_*MT*_), seal (*R*_*seal*_), and *C*_*MT*_ represents the capacitive components for the MT surface (see Text for details). The holding potential (Command voltage, *V*_*cmd*_) and tip potential (*V*_*tip*_) are different, as expected for the loose patch configuration (See Methods). (**d)** Time series recording of a patched rat brain MT bundle under symmetrical KCl conditions (both pipette and bath), to which several holding (positive) potentials were applied as indicated in the Figure. Expanded tracings show the increase in amplitude and complexity of the spontaneous oscillations. (**e)** Time series of a recording held at 7 mV, showing changes in amplitude, spontaneous sudden death, and complete recovery of the oscillatory behavior in the absence of any changes in driving forces. (**f)** *Top panel*, Fourier power spectra obtained from (1) unfiltered current tracings from a free-floating pipette before attachment, (2) after attachment to the MT bundle, and (3) same sample after treatment with Taxol (10 μM). *Bottom panel*, 3D phase-space portraits from spectra shown on *Top*, indicating limit cycles for the attached sample under control conditions, and much reduced after Taxol treatment. Color code as in *Top Panel*. Delay time for first and second derivatives adopted for phase portraits was 1 ms.

To determine the electrical activity of rat brain MT bundles, the patch clamp technology was used as previously reported^13,14,19^. Bundles of brain MTs (Fig. 1a,b) were obtained by one cycle of depolymerization-polymerization as described^19,20^. The surface was approached with the patch pipette connected to a patch clamp amplifier as indicated in Fig. 1c. Experiments were conducted with small tipped (4 μm^2^) pipettes under symmetrical ionic conditions, with a “high K^+^ intracellular” solution in both the patch pipette and bathing solutions (see Methods). Different tubulin conformations were observed in the preparation, including MT sheets and bundles (Fig. 1a,b). Apposition of the pipette tip onto an MT bundle (Fig. 1b) only increased the seal resistance from 20.3 ± 1.3 MΩ to 53.25 ± 7.59 MΩ, n = 25, Median 36 MΩ, and a range of 11.4 M Ω to 170 MΩ, in contrast to the high seal resistance usually obtained with 2D MT sheets^19^. This approach rendered a type of loose patch configuration^21–24^, as it has been used in numerous occasions to explore ion channel activity and electrical behavior in various excitable preparations^25–27^. The equivalent circuit of the experimental setup is shown in Fig. 1c, where it is expected that the actual driving force, namely the holding potential (*V*_*cmd*_, command voltage) at the surface of the MT bundle would be reduced by a factor related to the voltage divider circuit, between the pipette, seal, and MT bundle intrinsic resistances. No attempt was made to correct the potential, except for driving the tip potential to zero mV prior to approaching the sample.

**Figure 2:**
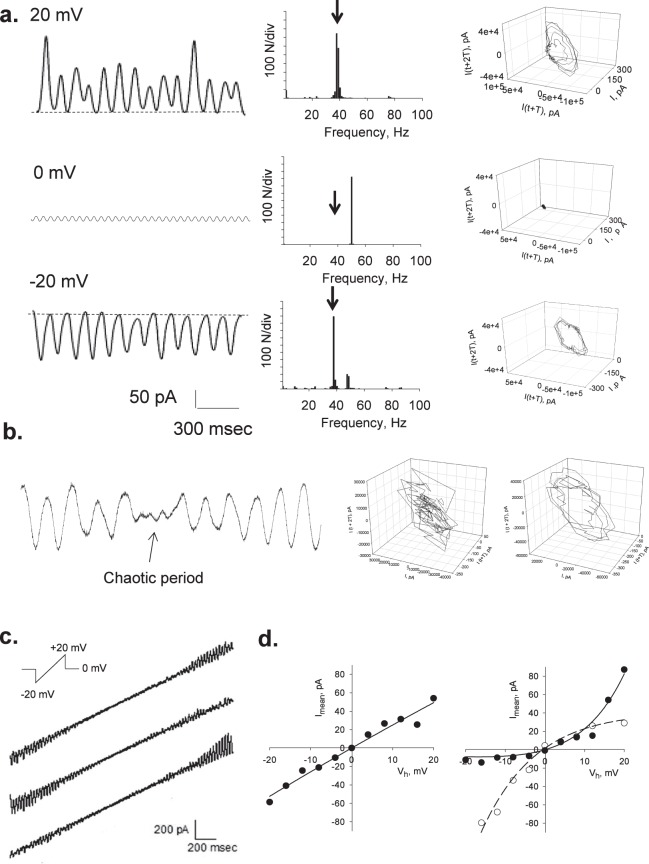
Current oscillations of voltage-clamped MT bundles. (**a)** Left. Representative tracings showing the oscillatory electrical behavior of an MT bundle at different holding potentials in symmetrical KCl. *Middle*. Linear-linear detail of Fourier power spectra obtained from unfiltered current tracings shown on the left, indicating a fundamental frequency of 39 Hz. No peak is observed at zero mV but instead appears a prominent 50 Hz signal due to line contamination. *Right*. 3D phase-space portraits showing monoperiodic limit cycles. Delay time for first and second derivatives adopted for phase portraits was 1 ms. (**b)** *Left*. Tracing showing the transition between non-chaotic and chaotic behavior, without any changes in driving forces. The chaotic (*Middle*) and non chaotic (*Right*) behaviors are shown in the 3D phase portrait. (**c)** Current oscillations under symmetrical KCl conditions were also obtained at instantaneous holding potentials applied from two-ms ramps between ± 20 mV, showing either linear (Top Panel) or both inwardly and outwardly rectifying properties in the same patch (Middle and Bottom panels, respectively). **(d)** The current-to-voltage relationships obtained from integrated, mean currents of individual experiments were highly linear (*Left*). However, longer time series elicited both strong inward and outward rectifications without any changes in driving forces (*Right*).

Sealed MT bundles displayed spontaneous electrical activity consistent with self-sustained electrical oscillations that responded directly to the magnitude of the stimulus (Fig. 1d). MT bundles were initially voltage-clamped at a holding potential of zero mV. Despite the symmetrical ionic conditions, electrical activity was clearly observed in all successful experiments (n = 22). The most frequently observed initial response consisted of strong bursts of oscillations that varied in both amplitude and frequency (Fig. 1d) in the absence of any external change in driving forces. A fundamental frequency of 29 Hz was observed only once (Fig. 1d, inset), which was similar to that of MT sheets at different holding potentials^19^. Most frequently however, the fundamental frequency rapidly shifted to 39 Hz (Fig. 1d Insets). The spontaneous oscillations showed changes in regime, including sudden death and recovery without any changes in driving forces (Fig. 1e). The MT bundle intrinsic electrical activity was confirmed by Fourier spectral analysis of time series output currents from the free-floating pipette before, after attachment, and subsequent Taxol (10 μM)-inhibition of the MT bundle oscillations (Fig. 1f). Clearly, the unattached pipette showed a white noise spectrum while the MT bundle attached spectra display the expected peaks of relevant frequencies in oscillatory behavior. Highly synchronized electrical oscillations were observed at different holding potentials of control, sealed MT bundles (Fig. 2a).

Although differences in the oscillatory regimes were often found, the fundamental frequency of 39 Hz was confirmed by Fourier transformation (Fig. 2a, Middle). 3D phase portraits constructed with the time delay method showed monoperiodic limit cycles (Fig. 2a, Right). The organized oscillations, however, shifted spontaneously to chaotic periods with more complex behaviors (Fig. 2b). Electrical currents were also obtained with voltage ramp protocols (Fig. 2c). Under these conditions, peak oscillatory deflections represented a maximal change in conductance of 12.48 ± 3.74 nS (n = 4, p = 0.001). A linear mean conductance of 2.54 nS was obtained from voltage step protocols in symmetrical 140 mM KCl (Fig. 2d, Left). Longer time series however, elicited both inward and outward rectifications in conductance (Fig. 2d, Right). The rectifying behavior was well approximated by the 2S3B energy model, as previously reported for MT sheets^19^.

**Figure 3:**
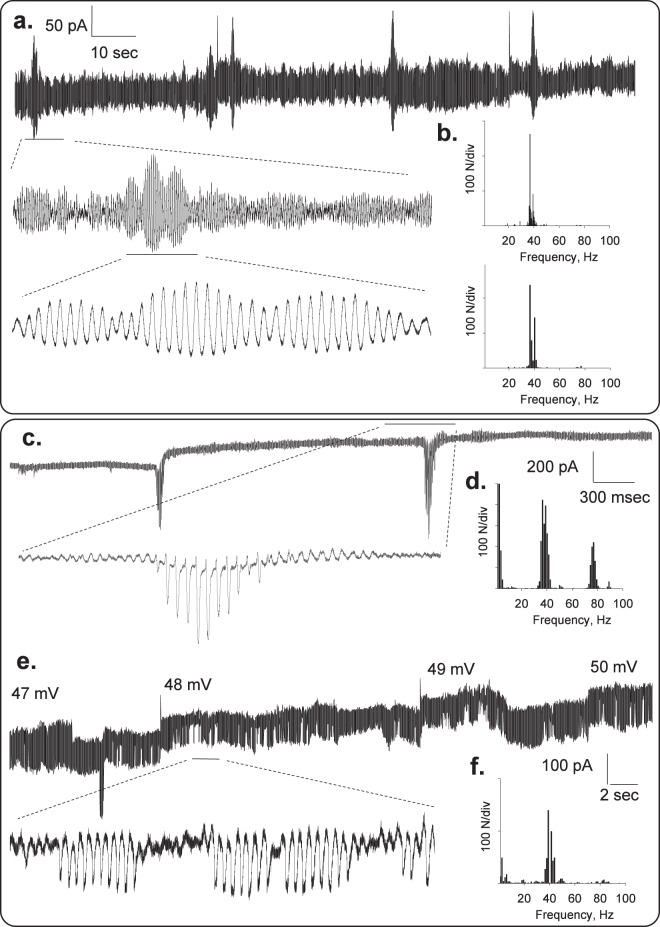
Spontaneous changes in oscillatory behavior. The oscillatory behavior of the patched MT bundles showed various spontaneous regimes without any external changes in driving force. Periodic changes in the amplitude of the oscillations showed fractal “envelopes” of increasingly lower frequency and different oscillatory regimes **(a**,**c**,**e)**. Power spectra for respective tracings are shown, where the 39 Hz fundamental frequency is present in all cases (**b**,**d**,**f**).

Periodic changes in the amplitude of the oscillations showed fractal envelopes (Fig. 3a) with a fundamental frequency of 39 Hz (Fig. 3b). Higher and lower frequencies were also observed (Fig. 3c–f), regardless of the applied holding potential.

**Figure 4:**
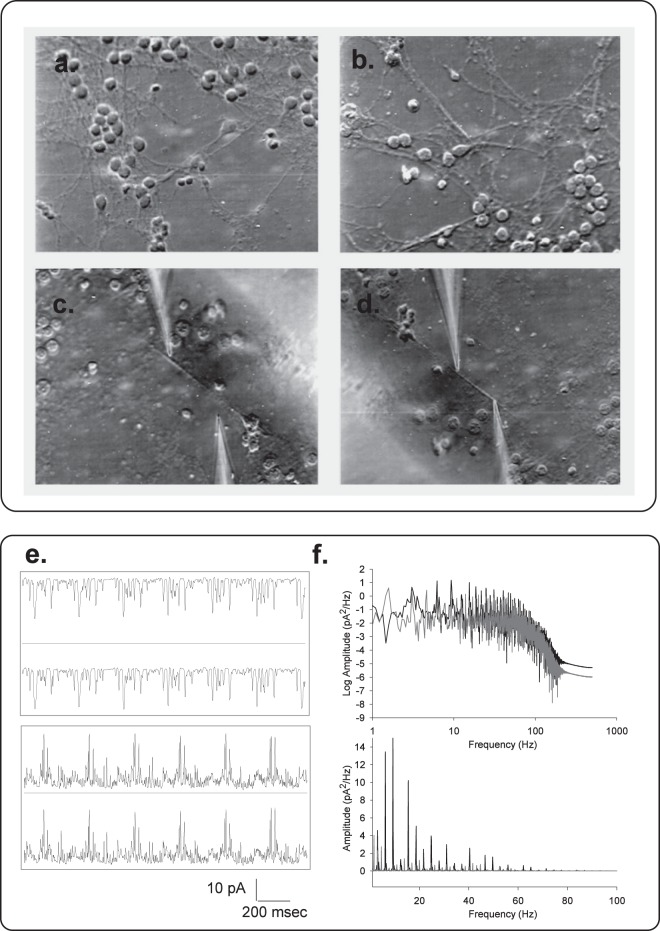
Electrical oscillations and coupling in membrane-permeabilized neurites. (**a**,**b)** Images of one-week old cultured hippocampal neurons(x20), before and after cell membrane permeabilization by addition of 1% (v/v) Triton X to the bathing solution. (**c**,**d)** Permeabilized neurites before and double patching with patch-clamp pipettes as shown in the actual locations of electrical recording. (**e)** Spontaneous electrical oscillations observed in one pipette and mirror images collected on the second pipette. Time series were obtained for a ±1 mV bias applied to one of the pipettes, as shown on the Top and bottom tracings, respectively. (**f)** Power spectra of four times the length of the tracings shown on (**e)**, for the positive (Black) and negative (Gray) biased signals, respectively. Linear-Linear plots for respective spectra are also shown (Bottom). Raw data were Gaussian-filtered at 50 Hz for display purposes only. Data are representative of three independent experiments.

To further prove the existence of electrical oscillations in MT bundles, electrical activity was also sought by direct measurements in neurons. For these experiments, adult mouse hippocampal neurons were prepared and cultured for over a week, as previously reported^28,29^, with modifications, to establish interconnecting neurites (Fig. 4a). Cells were then membrane-permeabilized by addition of Triton-X (Fig. 4b–d) to the bathing solution that allowed access to the cytoskeleton. A double-patch methodology was then applied as we have previously reported for isolated MTs^13,14^. Electrical oscillations were observed under symmetrical conditions, with an “intracellular” solution in both, the patch pipettes and bathing solution, by biasing one of the pipettes (stimulus) by one or several mV, and collecting electrical currents from both pipettes placed several microns from one another (Fig. 4d). Low frequency electrical signals were observed on both ends of the neurite (Fig. 4e,f), which displayed different frequencies and complex behaviors, depending on the polarity of the bias. Mirror images in both pipettes indicated a transmission of the electrical oscillations along the length of the neurite cytoskeleton.

## Discussion

The data in this report demonstrate that rat brain MT bundles generate electrical oscillations without any external stimulation. Holding potentials as small as 1 mV induced large changes in the cytoskeletal conductance. The oscillatory response was similar but richer than that we recently reported for 2D MT sheets of different origins^19^. Although the electrical signals of the MT bundles were observed without reaching “tight seal” conditions, no attempts were made to correct for this “loose patch” configuration^23^, because the expected response would only decrease the linear contribution to the driving force (see Methods), as the seal resistance would act as a dissipation sink (Equation 1), without a major contribution to the waveforms. Actually, the condition used in this study was much less invasive than that observed under “tight seal” conditions^19^, which also rendered highly stable electrical oscillations. The data should be considered qualitatively, but clearly indicated highly complex behaviors that were extremely sensitive to local changes in the electrochemical potentials used to collect the electrical signals. The electrical response of the MT bundles depended on both, the magnitude and the polarity of the electrical stimulus. Mean currents obtained with voltage step protocols were often linear, although spontaneous changes in amplitude of the cyclic regimes were observed as well. MT bundles also elicited highly synchronized trains of current oscillations that mimicked the response observed with action potentials. Thus, the data are most consistent with the fact that the surface of the MT bundle would behave as an ion-selective barrier, capable of generating highly synchronized, self-sustained electrical oscillations, even richer than those we recently observed in 2D MT sheets^19^. It is expected that these electrical oscillations would, in turn, generate highly dynamic electric fields emanating from the entire MT structure. To give credence to a physiological function of this electrical behavior, we also explored the bare cytoskeleton of membrane-permeabilized neurites of adult mouse hippocampal neurons, which also displayed electrical oscillations. Further, the neuronal cytoskeleton was also capable of axially transmitting the endogenously generated signals endorsing the transmission line behavior of MTs we previously described^13–16^. Thus, the electrical behavior of brain MTs may actually be an important endogenous property that would play a relevant role in neuronal function. This electrical response could also depend on their location and specific geometry. Axonal MT bundles for example, are more loosely packed than those formed in non-neuronal cells^30^, thus possibly rendering distinct functional properties.

Previous evidence, raised from combined AFM imaging and SPM mapping of *in vitro* assembled MTs bundles showed a strong linear correlation for all SPM distances, suggesting a limiting different surface potential at distances longer than 400 nm^10^. Based on the fact that the MT wall could be envisioned as a structural sandwich of negative charges on either side facing adsorbed ions (space charge) from the bulk solution^31^, several transmural capacitors in series would have to be charged properly to allow ions through. The empirical parameters obtained by SPM, in combination with the AFM topological features of the MT bundles would suggest that several Debye lengths are required to dissipate the gradient^10^, which is consistent with the standing gradients generated by the electrical activity of these MT structures. In such scenario, a positive gate voltage relative to ground would force bulk cations to be injected into a buffer zone “de-doping” the gate region on one side, and attracting counterions on the opposite one thus allowing trans-MT electrodiffusional currents. Ion fluxes would then turn the circuit back to its previous state, generating an oscillatory cycle. The gating of the oscillatory mechanism would require that the applied voltage through the saline decreases the fix charge density at the outer layer of the MT bundle, a phenomenon that could be conceptually equivalent to “de-doping” in electrochemical devices^32^. The capacitive current generated by the gate would then trigger the nanopore conductance electrodiffusional circuit. The electrical model would also require that once “gated”, ionic conduction through the nanopores will strictly depend on the electrochemical gradient of the permeable ions, and the frequency of the oscillations that will control the magnitude of the change in conductance. Thus, this permeable pathway would be formed by the channel-like conduits formed by the electrostatically-induced vibrations of adjacent αβ tubulin heterodimers acting as electrical oscillators that allow the electrodiffusional ionic transport. This hypothesis is supported by the fact that the oscillatory phenomena of the MT bundles showed certain degree of sidedness. A sudden change in polarity would drive more “chaotic” cyclic behaviors. In this scenario, the various nanopores would then oscillate at intrinsic frequencies that can further synchronize, generating different, more or less complex behaviors, including amplitude modulation expected from networks of interconnected electrical oscillators.

There are a number of potential implications for the behavior of brain MT bundles as electrical devices, which will certainly provide to as yet unknown novel features in neuronal function and regulation. It is tempting to postulate, for example, that the electrodynamic properties of MTs may provide a suitable explanation for other, better known MT-supported phenomena such as fast axonal transport^33^. MT-supported electrical amplification, for example, may provide a novel means for directionality in MTs. Axonal MTs are highly polarized, while MTs in dendrites have both plus- and minus- ends pointing outward^34^. Particularly concerning neuronal function, however, the presence of distinct MT-induced oscillatory currents may be critical to the gating and regulation of cytoskeleton-coupled excitable ion channels. Both, glutamate^35^ and NMDA receptors^36^, for example, bind tubulin, and the connection between cardiac L-type Ca^2+^ channels and mitochondria is also mediated by MTs^37^. Direct interactions between ion channels such as TRPP2^38^ and TRESK^39^ and electrically active MTs may also modulate local electric fields that will contribute to their gating processes.

Electrical oscillations by MT bundles may also play a role in other cell functions. Long-distance recruitment of molecules^40^ could be regulated by coherent MT oscillations as previously postulated by Fröhlich’s theory, where energetically stimulated oscillators would induce coherent excitations^41^. This is particularly relevant in intracellular physiology as electrodynamic but not electrostatic interactions would exert long distance effects without screening^12^. Further, different MT structures may also have distinct properties relying on their complexity and specific geometry. Centrosomes, which are relevant to cell division, contain two perpendicular centrioles each composed of nine MT triplets. Local electric fields generated by electrical oscillations within centrioles, would have distinct electrical properties that may aid in the mechanisms of centrosome separation and bipolar spindle body assembly. Motor proteins such as kinesins and dyneins that aid in centrosome separation and spindle assembly, require a close proximity to MT structures to efficiently generate the required pulling forces. Thus, electrical activity by MT bundles may provide an efficient means to regulating these interactions.

In summary, the data in this report demonstrate that rat brain MT bundles are electrically active. The electrical oscillations generated by MT bundles may provide a novel signaling mechanism relevant to various other cell functions, not only helping the transfer of electrical information in neurons, but also the control of cell division, and the transport of cargo in MT-driven organelles such as axons, cilia and flagella. These electrical oscillations may be at the center of intracellular electric fields in the brain, and may help address open questions of higher brain functions, including the molecular aspects of anesthesia^42^, and the issue of consciousness^43^. Electrical oscillations by MT bundles open a novel field of biological signaling, particularly in neuron function.

## Methods

### Preparation of MT bundles from rat brain

For the present studies, rat brain MTs were obtained as recently described^19,20^. Briefly, brain tissue was homogenized in a blender with buffer containing in mM: 100 MES (pH 6.4), 2.0 EGTA, and 1.0 MgSO_4_, and further homogenized with a Teflon-in-glass homogenizer, and then centrifuged at 100.000 *g*. Finally, GTP (1.0 mM) was added to the supernatant, and incubated for 24 h. The bundles of MTs were identified under DIC and immunochemistry (anti-α tubulin antibody, Santa Cruz Biotechnology, Dallas TX) with an Olympus IX71 fluorescence inverted microscope. Samples were kept frozen at −20 °C until further use. To initiate the experiments, an MT sample was deposited onto a silanized glass surface, treated with a solution containing APTES (3-aminopropyl-triethoxysilane), as recently reported^19^. Briefly, freshly prepared APTES (0.1%, v/v, 02154766, MP Biomedicals) in distilled water was applied and dried onto a clean glass coverslip for 10 min before seeding the MT preparation.

### Immunochemical labeling of MT samples

MTs samples were labeled to visualize α-tubulin. The antibody raised from rabbit against amino acids 149–448 of human α-tubulin was obtained from Santa Cruz Biotechnology Inc (H-300, sc-5546) and used at 1:500 dilutions as previously reported^19^. The secondary antibody used for tubulin staining was bovine anti-rabbit IgG-R (sc-2367, Santa Cruz Biotechnology Inc, CA) used at a 1/1000 dilution. Samples were viewed under an inverted Olympus IX71 microscope connected to a digital CCD camera C4742-80-12AG (Hamamatsu Photonics KK, Bridgewater, NJ). Images were collected with the IPLab Spectrum (Scanalytics, Viena, VA) acquisition and analysis software, running on a Dell-NEC personal computer.

### Electrophysiological data acquisition and analysis of MT bundles

The electronic setup to obtain electrical recordings from voltage-clamped MT bundles consisted of a conventional patch clamping amplifier (Axopatch 200B, Molecular Devices, Sunnyvale CA), directly connected to the MT sample via a saline-containing patch pipette, as recently reported^19^. Experiments were conducted with an “intracellular” KCl solution containing, in mM: KCl 140, NaCl 5, EGTA 1.0, and Hepes 10, adjusted to pH 7.18 with KOH. Briefly, MT bundles were identified in the MT preparation (Fig. 1b), approached by the patch pipette and sealed by light positive pressure of the tip onto the surface (Fig. 1a,c). Seal resistance was obtained by imposing 1 mV square pulses. The pipette tip in solution most often rendered resistances in the order of 5–15 MΩ, as indicated by current deflection and application of Ohm’s law. Patch pipettes were made from soda lime capillary tubes (Biocap, Buenos Aires, Argentina) with 1.25 mm internal diameter and a tip diameter of ~4 μm. Voltage clamp protocols included steady steps at various holding potentials (gap-free protocol), trains of 1500 ms pulses at different holding potentials from zero mV, and 1500 ms ramps within the same voltage range. Electrical signals were acquired and filtered at 10 kHz, digitized with an analog-digital converter (Digidata 1440 A, Molecular Devices) and stored in a personal computer with the software suite pCLAMP 10.0 (Molecular Devices), also used for data analysis. Sigmaplot Version 10.0 (Jandel Scientific, Corte Madera, CA) was used for statistical analysis and graphics.

### Loose-patch clamp configuration

The loose-patch-clamp configuration is analog to the cell-attached voltage-clamp method, where voltages applied to the pipette affect the surface under its opening. The main difference between techniques lies in seal resistance considerations. In tight-seal cell-attached patches, the seal resistance is very large (≥1 GΩ), such that very small currents are expected through the seal, thus making the pipette tip potential close to the value of the applied holding potential. Loose patches, in contrast, have considerably smaller seal resistances (MΩ range), such that significant currents flow through the seal affecting the tip potential (Fig. 1c).

In our preparation, the command voltage (*V*_*cmd*_), which is the holding potential applied through the amplifier, will not be the same to the tip potential “seen” by the MT surface. The difference will depend on the magnitude of the seal resistance. A simple model circuit analysis (Fig. 1c) shows that when having a low seal resistance, the voltage at the pipette tip (*V*_*p*_) will be given by

1 $${V}_{tip}={V}_{cmd}(\frac{{R}_{seal}}{{R}_{seal}+{R}_{pip}})={V}_{cmd}\times B$$

where *R*_*pip*_, *R*_*seal*_, and *V*_*cmd*_ are the pipette resistance, seal resistance, and command voltages, respectively. Under such conditions, where *R*_*seal*_ is of the order of magnitude of *R*_*pip*_, the voltage at the pipette tip will thus be reduced from the command voltage by a factor of *B* (note that when *R*_*seal*_ ≫ *R*_*pip*_, then *V*_*pip*_ *≈ V*_*cmd*_, as in the tight-seal case).

### Other current analyses

Unless otherwise stated, electrical tracings shown throughout the study were unfiltered data. Average currents at various holding potentials were obtained by integration of one-second tracings, and expressed as mean ± SEM values, where (n) represented the number of experiments analyzed for a given condition. Power spectra of unfiltered data were obtained by Fourier transform with a subroutine from Clampfit 10.0. Limit cycles were constructed by the time delay (*τ*) approach from the unfiltered tracings, where the lag time *τ* was chosen arbitrarily at 2*f*, where *f* was the sampling frequency of data acquisition. Three dimensional phase space diagrams were constructed in Sigmaplot 10.0.

### Energy modeling of the MT bundle conductance

The nature of the time and voltage-dependent changes of the spontaneous oscillations suggested that the constant field equation of ionic conductance would be unable to model the ionic conductance. Thus, data were fitted with an Eyring multi-barrier rate theory model accounting for the intrinsic rectification and the free energy profile for ion transfer with a three-barrier-two-site (2S3B) minimal conductance model that supports multiple occupancy and saturation, as recently reported^19^. Briefly, the model included six energy parameters: three peak energies (*G*_12_, *G*_23_ and *G*_34_), two well energies (*G*_2_ and *G*_3_), and three electrical distances (*d*_1_ to *d*_3_), that represent the fraction of the electric field energetically separating peaks and wells. An interaction parameter, *A* = F_out_/F_in_, was also included to represent ion-ion interactions, where F_in_ and F_out_ are the repulsion factors inside and out the conductive pores, respectively, after ion occupancy. For high activity ranges^44^ the current *I* may be approximated by equation (2):

2 $$I=zFQ\exp (-{G}_{23}+{G}_{3}+{G}_{2})A\{\frac{\exp [({d}_{2}+2{d}_{1}){V}_{h}]}{{[{S}^{+}]}_{bath}}-\frac{\exp [({d}_{2}+2{d}_{3}){V}_{h}]}{{[{S}^{+}]}_{pipette}}\}$$

where [*S*^+^]_bath_ and [*S*^+^]_pipette_ are the concentrations of permeable ion in the bath and the pipette, respectively, *V*_*h*_ is the holding potential, *F* is the Faraday constant, and *Q* represents a term enclosing the rate constants between pore states^44,45^.

### Dissociation and culture of hippocampal neurons

Adult mouse hippocampal neurons were obtained as originally described, with modifications^28,29^. Briefly, C57Bl mice of 5–7 weeks old were killed by cervical dislocation and decapitated according to IACUC guidelines. Hippocampi were dissected out into ice-cold Ca^2+^-free medium Hibernate A (BrainBits, Springfield, IL), and minced into small pieces. Flushing a few times through a fire-polished Pasteur pipette further dispersed the tissue. The supernatant containing the dissociated hippocampal neurons was centrifuged at 200 *g* for 1 min. The cell pellet was resuspended in NbActive4 medium (BrainBitz, Springfield, IL) and seeded onto poly-L-lysine-coated glass coverslips. Hippocampal cells were incubated at 37 °C, in a wet incubator gassed with 5% CO_2_, 20% O_2_. Hippocampal neurons were kept in culture for up to two weeks with NbActive 4 medium changes every five days

### Electrophysiology of membrane-permeabilized neurites from cultured hippocampal neurons

One-to-two week cultured hippocampal neurons were washed in an extracellular saline solution containing (in mM): 135 NaCl, 5.0 KCl, 1.2 CaCl_2_, 10 Hepes, pH 7.4, to eliminate culture medium and then viewed under DIC (x20). The bathing solution was then replaced with an intracellular saline, containing (in mM): 135 KCl, 5.0 NaCl, 10 Hepes, 1.0 EGTA, pH 7.4. Electrical signals from the neurites were recorded under voltage clamp conditions with a Axopatch 200B amplifier (Axon Instruments) and a Dagan 3900 A amplifier, respectively. The signals from both pipettes were digitized with a 1440 A Digidata (Axon Instruments) and analyzed with pClamp10.0 software (Axon Instruments). Electrical signals were Gaussian filtered at 50 Hz for display purposes. Data were viewed and analyzed with pClamp 10.

### Ethical statements

All experimental protocols were approved by the Ethics Committee from the Facultad de Odontología, Universidad de Buenos Aires (approved protocol number 014/14, UBA resolution 0082153/2013). All methods were carried out in accordance with approved guidelines.
